# QuEChERS analytical approach for monitoring endocrine-disrupting chemicals in human urine

**DOI:** 10.1007/s00216-025-06099-1

**Published:** 2025-09-24

**Authors:** S. Callejas-Martos, E. Eljarrat

**Affiliations:** Environmental and Water Chemistry for Human Health (ONHEALTH), Institute of Environmental Assessment and Water Research (IDAEA)-CSIC, Jordi Girona 18-26, 08034 Barcelona, Spain

**Keywords:** Human biomonitoring, Metabolites, Organophosphate esters, Parabens, Phthalates, Target analysis

## Abstract

This study introduces an analytical method for the determination of endocrine-disrupting chemicals (EDCs) in human urine samples, employing QuEChERS as a clean-up step for the first time in this biological matrix. Sample preparation was rigorously optimized through the evaluation of sample and final dilution volumes, balancing the need to reduce matrix interference without sacrificing sensitivity. High-performance liquid chromatography coupled with a triple quadrupole-time-of-flight mass spectrometer (HPLC-QTOF) was employed, enabling the detection of 13 target analytes (five OPEs, five phthalates, and three parabens’ metabolites). Chromatographic separation was achieved in 16 min, and mass spectrometry parameters were fine-tuned to maximize signal sensitivity. The method was validated by assessing parameters such as selectivity, linearity, precision, accuracy, and sensitivity. Linearity of response was demonstrated for all of the compounds (r^2^ > 0.99), with method detection limits of 0.01–0.33 ng/mL and limits of quantification of 0.03–1.08 ng/mL. Accuracy ranged from 67 to 99% and inter- and intra-day precision were under 20%, for the majority of the analytes. The validated method was then applied to 39 human urine samples, confirming its reliability and practical utility for monitoring EDC exposure in urban populations. The highest concentrations were found in parabens, followed by phthalates and finally OPEs, with median values of 117, 43.5, and 25.4 ng/g of creatinine, respectively. The detection of all three EDC groups in 100% of the samples underscores the importance of this comprehensive analytical approach being capable of simultaneously assessing these contaminants in human biomonitoring studies.

**Graphical Abstract:**

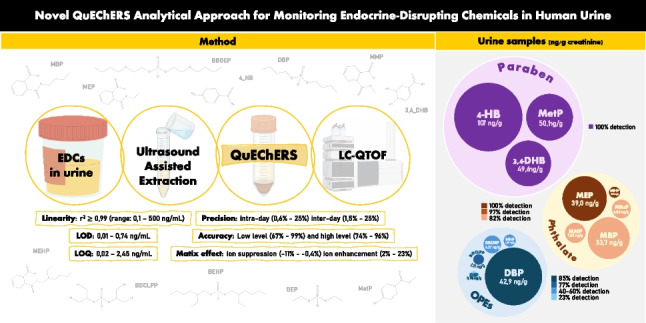

**Supplementary Information:**

The online version contains supplementary material available at 10.1007/s00216-025-06099-1.

## Introduction

Endocrine-disrupting chemicals (EDCs) have become a focal point of research due to their ability to mimic natural hormones [1, 2]. This structural similarity allows them to disrupt various biological processes, interfering with the endocrine system, and potentially leading to adverse health effects. Studies [3, 4] have revealed that EDCs impact progesterone and testosterone levels, and their alteration can result in metabolic problems for both sexes.

Among this wide group of EDCs, organophosphate esters (OPEs) are particularly noteworthy given their widespread application as flame retardants in textiles, plastics, paints, foams, lubricants, and electronics products [5]. Additionally, OPEs Like 2-ethylhexyl diphenyl phosphate (EHDPP) are used as plasticizers in food packaging [6], and triphenyl phosphate (TPHP) is commonly found in nail polish [7]. The extensive use of these chemicals has led to their dispersion into indoor and outdoor environments [8, 9], raising concerns about their potential impact on human and ecological health. OPEs are suspected to have various toxic effects, including mutagenic, carcinogenic, and neurotoxic effects, apart from the endocrine-disrupting properties [10].

Phthalates represent another major group of EDCs, widely employed across numerous applications. These synthetic organic chemicals are commonly added to plastics to enhance flexibility, transparency, durability, and elasticity. Their application varies based on their molecular weight. High molecular weight phthalates are found in polyvinyl chloride (PVC) plastics. Low molecular weight phthalates are used in personal care products, varnishes, and coatings [11]. Phthalates have been previously linked with reproductive and metabolic health issues [12, 13]; several phthalates, including di(2-ethylhexyl) phthalate (DEHP), diisononyl phthalate (DiNP), di-n-butyl phthalate (DnBP), and butyl benzyl phthalate (BBzP), have been restricted in children’s toys and childcare products [14]. Moreover, their classification as a presumed human reproductive toxicant (Category 1B) has led to the ban of DEHP, DnBP, diisobutyl phthalate (DiBP), and BBzP in cosmetic applications under European regulation 2004/93/EC [15].

Parabens constitute another group of EDCs. They are synthetic alkyl esters of p-hydroxybenzoic acid, widely used as preservatives in consumer products, including packaged foods, cosmetics, and personal care items [16–18]. Their popularity is attributed to their antibacterial and antifungal properties, cost-effectiveness, and high chemical stability [19]. Among the commonly used parabens are methyl (MetP), ethyl (EtP), propyl (PrP), butyl (BuP), benzyl (BzP), and heptyl parabens (HepP) [17]. Despite their reputation for low toxicity, recent evidence suggests that their accumulation in the human body—particularly in adipose tissue—may contribute to endocrine and reproductive toxicity, potentially promoting obesity [18]. Studies have further identified 4-hydroxybenzoic acid (4-HB) and 3,4-dihydroxybenzoic acid (3,4-DHB) as common metabolites of several parabens in both animals and humans [17].

All these groups of EDCs are added to products as non-covalently bound additives, which allows them to leach easily into the environment. This has led to their widespread presence in water, air, soil, sediment, and even indoor dust, leading to extensive human exposure. Given the widespread presence and potential health risks of these synthetic compounds, human biomonitoring (HBM) [6, 18] has become a crucial method for assessing their total exposure across all possible routes (i.e., inhalation, ingestion, and dermal contact).

Once EDCs reach the human body, they have a short half-life and are rapidly metabolized into bioactive monoesters, which are then excreted via urine in either free or glucuronide-conjugated forms [12, 20, 21]. Some EDCs are further transformed into secondary oxidized metabolites, also eliminated via urine. So, the EDC metabolites’ presence, together with their high availability, ease of collection, and non-invasive nature, make urine one of the principal matrices used in HBM studies [22, 23]. However, the complexity of urine as a biological matrix presents unique challenges [24]. One of the main ones is the huge presence of endogenous matrix compounds, present at concentrations far higher than the target synthetic chemicals. Additionally, the physical and chemical properties of endogenous substances often overlap with those of the target metabolites, complicating the removal of these endogenous compounds without losing the targets of interest.

To address these challenges, careful sample preparation and analysis procedures are essential. Common urine sample preparation approaches include dilution, protein precipitation, and extraction techniques such as liquid–liquid extraction (LLE) and solid-phase extraction (SPE) [18, 22]. For the analysis, liquid chromatography coupled with high-resolution mass spectrometry (LC-HRMS) has become the preferred technique, providing the sensitivity needed to detect and quantify the exogenous chemicals and their metabolites at increasingly low levels [25, 26]. Many of these techniques have been used previously to develop analytical methods for biomonitoring OPEs, phthalates, and parabens in urine, typically focusing on each chemical family separately [27]. Although these approaches are effective, conducting three independent analyses requires larger sample volumes—a critical consideration in human biomonitoring studies where sample availability is often limited. Additionally, separate analyses increase costs associated with reagents, instrumentation, and processing time.

This study presents a more efficient alternative by simultaneously identifying metabolites from all three EDC families (OPEs, phthalates, and parabens) using a newly developed target screening protocol. This method is based on the use of QuEChERS, which requires a lower sample volume, a less extensive extraction procedure, and uses the most cost-effective material. To the best of our knowledge, no previous studies have employed the QuEChERS method for the determination of these specific compounds in urine. The objectives of this work were [1] to optimize an LC-HRMS/MS analytical method for the identification of three groups of EDCs in human urine samples and [2] to apply the proposed method to a batch of human urine samples from adults and teenagers (*n* = 39).

## Materials and methods

### Chemicals and materials

Analytical standards were sourced from various suppliers: bis(1,3-dichloro-2-propyl) phosphate (BDCIPP), dibutyl phosphate (DBP), bis(2-butoxyethyl) phosphate (BBOEP), diethyl phosphate (DEP), bis(2-ethylhexyl) phosphate (BEHP), monomethyl phthalate (MMP), monoethyl phthalate (MEP), and labelled isotopes d_10_-BDCIPP, d_18_-DBP, d_4_-MMP, and d_4_-MBP were obtained from Toronto Research Chemicals (Toronto, ON, Canada). Monobenzyl phthalate (MBzP) and mono(2-ethylhexyl) phthalate (MEHP) were purchased from Dr. Ehrenstorfer (Augsburg, Germany), while monobutyl phthalate (MBP) was acquired from Merck (Darmstadt, Germany). The isotope-labeled compound ^13^C_6_-p-Hydroxybenzoic acid (^13^C_6_−4-HB) was obtained from Cambridge Isotope Laboratories Inc. (Andover, MA, USA). Additional chemicals, including 4-HB, 3,4-DHB, and MetP, were also used (Table S1).

Creatinine and β-glucuronidase deconjugation enzymes (lyophilized powder from *Escherichia coli*, with activity > 20,000 units/mg protein) were procured from Sigma-Aldrich (Bornem, Belgium). QuEChERS salt (SALT-Kit-AC2) was purchased from Bekolut (Bruchmühlbach-Miesau, Germany).

Potassium phosphate dibasic trihydrate (≥ 99.0%), ammonium acetate, and formic acid (≥ 98%) were also obtained from Sigma-Aldrich (Bornem, Belgium). Reagents for trace analysis, including ammonium acetate, formic acid, water, and methanol, were supplied by Merck (Darmstadt, Germany). Acetonitrile for trace analysis was purchased from Fisher Scientific Chemical, and methanol solvent was sourced from J.T. Baker (Centre Valley, PA, USA).

### Optimization of extraction process

Analyzing biological matrices such as urine is inherently challenging due to their complex composition, which can lead to significant matrix effects. Therefore, optimizing the extraction methodology requires striking a balance between achieving high sensitivity and reducing matrix effects. Two sample volumes, 2 and 5 mL, were evaluated to determine the optimal sample size for extraction. Then, the final dilution volume was also optimized. The extracts obtained through the sample extraction procedure were diluted to final volumes of 100, 500, and 1000 µL. Therefore, six different combinations of sample volume and final dilution were tested (2 mL–100 µL; 2 mL–500 µL; 2 mL–1000 µL; 5 mL–100 µL; 5 mL–500 µL; 5 mL–1000 µL). The matrix effect was quantitatively assessed by comparing the signal of each analyte in the urine matrix to its signal in methanol, allowing for the determination of the optimal conditions for minimal matrix interference.

### Final extraction method

The optimized analytical methodology uses 2 mL of urine sample. A homogeneous aliquot of the urine was spiked with a mixture containing 20 ng of each internal standard (IS): d_10_-BDCIPP, d_18_-DBP, d_4_-MMP, d_4_-MBP, and ^13^C_6_−4-HB. To this, 1.5 mL of phosphate buffer (1 M, pH 6) was added. Subsequently, 100 μL of β-glucuronidase from *Escherichia coli* (prepared as a 2 mg/mL solution in phosphate buffer) was introduced to facilitate enzymatic deconjugation. The mixture was then vortexed gently and incubated at 37 °C for 2 h. Enzymatic activity was stopped by the addition of 100 μL of formic acid.

The extraction of EDC metabolites was conducted using ultrasonic-assisted liquid extraction. This involved adding 4 mL of acidified acetonitrile (containing 1% formic acid) to the sample. The tubes were vortexed for 30 s and then placed in an ultrasonic bath (Selecta, Barcelona, Spain) at room temperature for 20 min. For the first time in urine sample analysis, a QuEChERS purification method was applied, based on a previous study [28] designed for the analysis of 360 toxicologically relevant pollutants in blood samples. Specifically, 1.5 g of SALT-KIT-AC2 from Bekolut (composed of 1200 mg anhydrous magnesium sulphate and 300 mg sodium acetate) was added to the mixture. The tubes were then vortexed for 30 s and manually shaken vigorously for 1 min. Following this, the samples were centrifuged at 4000 rpm for 10 min at 20 °C using a centrifuge 5810R (Eppendorf). The supernatant was carefully collected and evaporated under a gentle nitrogen stream to approximately 1 mL. If necessary, the remaining samples were centrifuged again, and the supernatants were evaporated to dryness before being reconstituted in 500 µL of methanol.

To minimize the risk of contamination during the extraction process, plastic equipment was completely avoided. Instead, glassware was used and underwent a thorough cleaning procedure. First, the glassware was washed with ultrapure water, ethanol, and acetone; this was followed by thermal treatment at 380 °C and a final rinse with solvent immediately before use. Each batch also included a blank control sample to detect and account for any additional background contamination.

### HPLC–MS/MS analysis

The analysis of EDCs was conducted using a liquid chromatography (LC) equipped with a Bruker Elute Pump HPG 1300 and coupled to a QTOF Impact II (Bruker, Bremen, Germany). Chromatographic separation was performed at 30 °C on a Purospher® STAR RP-18 end-capped HPLC column (2.1 × 150 mm, 2 µm particle size) with a SecurityGuard™ ULTRA Cartridge (UHPLC C18 2.1 mm) from Phenomenex serving as a guard column. The mobile phase consisted of 10 mM ammonium formate (NH_4_F) with 0.1% formic acid in water (phase A) and 10 mM NH_4_F in methanol (phase B). The injection volume was set at 10 µL with a flow rate of 0.3 mL/min. An optimized linear gradient was employed for the accurate identification of 13 native compounds and 5 IS. The gradient profile was as follows: 10% B initially and maintained for 30 s, increased to 90% B from 0.5 to 5 min, then increased to 100% B from 5.1 to 9 min, and kept at 100% B for 2 min, and finally re-equilibrated to 10% B from 11.1 to 12 min and maintained at 10% B for 4 more minutes.

Mass spectrometric analysis was performed using an electrospray ionization (ESI) interface in negative mode. The ESI parameters were configured as follows: drying gas temperature was maintained at 200 °C with a flow rate of 8.0 L/min; the probe gas temperature and flow rate were also set to 200 °C and 4 L/min, respectively. Nebulizer pressure was maintained at 3 bar. The capillary voltage was set to 2500 V, and the end plate offset was set to 500 V. Broadband collision-induced dissociation (bbCID) data acquisition mode was utilized for target screening. This mode involves two sequential full-scan events: the first scan at a low collision energy of 5 eV and the second scan at a high collision energy ramping from 5 to 30 eV, which provides comprehensive fragmentation data for all ions. MS spectra were recorded across a mass-to-charge ratio (*m/z*) range of 30–1000 Da, with a scan rate of 3 scans per second, achieving a frequency of 1 Hz in MS and 3 Hz in MS/MS. All urine samples were analyzed under these specified chromatographic and mass spectrometric conditions.

### Method validation

Method validation was conducted in accordance with the guidelines provided by the European Medicines Agency [29, 30]. The analytical performance of the method was evaluated in terms of selectivity, linearity, limit of detection (LOD), limit of quantitation (LOQ), intra-day and inter-day precision, accuracy, matrix effect, and carry-over. Due to the unavailability of blank urine samples for contaminants like EDCs, all analytical parameters were determined using urine pool samples.

The selectivity of the method was assessed based on the mass accuracy achieved by the QTOF instrument, using spiked pooled urine samples. Linearity was evaluated across a 0.1 to 5000 ng/mL concentration range. The linearity of the method was determined by calculating the coefficient of determination (r^2^) from a calibration curve constructed using spiked urine pool samples. The calibration curve was prepared with eight concentration points, each analyzed in triplicate, and included IS at a concentration of 50 ng/mL. When specific internal standards were unavailable, homologous ISs with similar structural properties or retention times were used (see Table 1).

**Table 1:** Analytical parameters for each target metabolite, including retention time (RT), exact mass-to-charge ratio (m/z), internal standard associated, linearity (expressed as correlation coefficient (r^2^) and range of linearity), sensitivity (LOD and LOQ), matrix effect and mass accuracy

	Acronym	RT (min)	*m/z* (M + H)^+1^	IS associated	Linearity (r^2^)	Range (min→max)	LOD (ng/mL)	LOQ (ng/mL)	Matrix effect (%)	Mass accuracy (|ppm|)
**OPEs**	BDCIPP	6.2	316.9071	d_10_-BDCIPP	0.9995	0.10**→**500	0.313	1.043	−10	1.58
	DBP	6.1	209.0943	d_18_-DBP	0.9999	0.10**→**500	0.010	0.033	13	0.96
	BBOEP	6.4	297.1467	d_18_-DBP	0.9997	1.00**→**500	0.013	0.043	−11	1.68
	DEP	2.9	153.0317	d_18_-DBP	0.9996	0.10**→**500	0.146	0.488	2.0	3.92
	BEHP	7.7	321.2195	d_18_-DBP	0.9994	0.10**→**500	0.110	0.368	13	1.56
**Phthalates**	MMP	5.0	179.0344	d_4_-MMP	0.999	0.10**→**500	0.166	0.555	10	1.12
	MEP	5.4	193.0501	d_4_-MBP	0.9993	0.10**→**500	0.216	0.719	−0.4	1.55
	MBzP	5.9	255.0657	d_4_-MBP	0.9992	50.0 **→**500	0.270	0.901	−2.9	1.57
	MBP	6.1	221.0914	d_4_-MBP	0.9998	0.10**→**500	0.055	0.183	−2.9	3.17
	MEHP	7.0	277.1440	d_4_-MMP	0.9997	0.10**→**500	0.325	1.083	10	1.44
**Parabens**	4_HB	4.8	137.0239	^13^C_6_4-HB	0.9994	0.10**→**500	0.130	0.433	2.5	1.46
	3,4-DHB	4.6	153.0188	^13^C_6_4-HB	0.9998	1.00**→**500	0.060	0.200	23	0.65
	MetP	5.8	151.0395	^13^C_6_4-HB	0.9993	0.10**→**500	0.013	0.042	12	1.32

LODs and LOQs can be calculated using multiple approaches. In this study, LODs and LOQs are calculated using signal-to-noise ratios of 3 and 10, respectively, at the lowest calibration point.

Precision and accuracy were evaluated using two levels of quality control (QC) samples: low concentration (LQC) at 30, 30, and 50 ng/mL, and high concentration (HQC) at 300, 300, and 500 ng/mL for OPEs, phthalates, and parabens, respectively. QC samples were prepared from pooled urine collected from multiple anonymous adult donors and then divided and spiked with native metabolites. Intra-day precision and accuracy were determined by conducting four independent measurements of each QC level within a single day. Inter-day precision was assessed by performing four independent measurements of each QC level over three separate days (*n* = 12). Precision was expressed as the relative standard deviation (RSD) of replicate measurements, while accuracy (expressed as %) was calculated based on the agreement between the measured and the spiked concentrations.

The matrix effect (ME) was assessed by comparing the peak area of analytes in spiked urine samples to those in pure solvent solutions (4:1 H_2_O/MeOH). The results were corrected using the matrix factor (MF) of the IS. Matrix effect (expressed in %) was calculated following Eq. 1, providing an indication of signal suppression or enhancement due to the presence of matrix components:

1 $$\text{ME}\left(\%\right)=\frac{\text{MF}}{{\text{MF}}_\text{IS}}=\left[\frac{\left(\frac{\text{Peak Area of Analyte in urine}}{\text{Peak Area of Analyte in Solvent Solution}}\right)}{\left(\frac{\text{Peak Area of IS in urine}}{\text{eak Area of IS in Solvent Solution}}\right)}-1\right]\times100$$

To evaluate carry-over effects, solvent blanks (4:1 H_2_O/MeOH) were analyzed immediately following the highest QC sample. Laboratory blanks and instrumental blanks (MeOH injected after every 5 real samples) were also analyzed throughout the procedure to monitor potential cross-contamination and carry-over.

### Creatinine analysis

Determining creatinine concentrations in urine samples is essential for correcting metabolite levels due to variations in urine dilution [31]. The creatinine levels were measured using LC–MS/MS, following a slightly modified version of the methodology proposed by Park et al. [31]. This modified method is simple, sensitive, and specific for urinary creatinine determination. Sample preparation involved diluting 10 μL of urine directly into 10 mL of methanol.

Analysis of creatinine was performed in an LC coupled to a triple quadrupole (QqQ) by Thermo Fisher Scientific (Waltham, MA, USA), with a heated-electrospray ionization (H-ESI) in positive mode. Chromatographic separation was achieved by injecting 10 μL of the prepared solution in a Purosphere Star RP-18 (125 mm × 0.2 mm, particle size 5 µm) column at a flow rate of 0.20 mL/min. The mobile phases used were water (solution A) and acetonitrile (solution B), both containing 0.1% formic acid. Chromatographic separation was performed under isocratic conditions with a gradient composition of 50% A, 50% B, for a total time of 4 min. Selected reaction monitoring (SRM) mode was employed, with two transitions monitored: 114 > 86 for quantification and 114 > 44 for confirmation. The decluttering potential (DP) was set at 43 V, with collision energies (CE) of 11.1 V and 12.1 V for the quantification and confirmation transitions, respectively. These parameters were fine-tuned by directly infusing a creatinine standard in full-scan mode into the MS. An external calibration method was used, with a five-point calibration curve ranging from 50 to 5000 ng/mL.

### Data analysis and statistical treatment

Post-acquisition data processing was performed using Compass DataAnalysis 5.0 and TASQ 2.1 software (Bruker Daltonics, Bremen, Germany). All urinary metabolite concentrations reported were adjusted for urine dilution by creatinine unless indicated otherwise. Concentrations below the LOD were substituted with a value of LOD/√2. Only metabolites with a detection frequency (DF) greater than 50% were included in statistical analyses.

### Application to real urine samples

A total of 39 urine samples were collected and analyzed in this study. These samples included those from 29 teenager volunteers, aged between 14 and 16 years, obtained with parental consent, and 10 adults (equally divided between males and females), aged 24 to 33 years. All participants resided in the urban area of Barcelona, Spain. Informed consent was obtained from all participants, detailing the purpose of the study and the procedures for urine sample collection. Samples were collected from the first-morning urine in specialized polystyrene containers (DELTALAB, S.L., Barcelona, Spain) and stored at−80 °C until analysis.

## Results and discussion

### LC–MS/MS optimization

The final chromatographic separation program, detailed in the “HPLC–MS/MS analysis”, section, was adapted from previously developed methodologies from the literature [32]. Several concentrations of ammonium formate and formic acid were tested to identify the most effective organic modifiers. For the majority of analytes, the optimal signal-to-noise ratio and peak area were achieved using 0.1% formic acid and 5 mM ammonium formate. These conditions provided both enhanced ionization and efficient separation, allowing for the complete separation of all thirteen target analytes within a 16-min run time (Figure S1). The chromatographic run achieved minimal overlapping peaks, with a well-distributed separation, particularly important for detecting low-level concentrations of analytes that could otherwise be obscured by high-abundance endogenous matrix compounds.

For mass spectrometry optimization, individual standard solutions of each analyte were used to characterize each analyte and determine their characteristic broadband collision-induced dissociation (bbCID) fragment ions, which were then compiled into an internal database for targeted screening. Subsequently, the electrospray ionization (ESI) source parameters were optimized using a mixed standard solution to achieve high sensitivity and strong signal intensity across all target compounds. The optimized parameters, including nebulizer gas pressure, drying gas flow rate and temperature, and capillary voltage, are detailed in the section.

### Sample preparation optimization

The optimal combination of sample and final volume was selected based on the comparison of the minimal matrix effects for each analyte while maintaining the maximum sensitivity possible (Table S2). The matrix effect and sensitivity were calculated as explained in the “Method validation” section.

In general terms, no significant differences in sensitivity were noted across the various tests; slight improvements in the LOD were observed for certain analytes when using higher sample volumes. For instance, the LOD for BDCIPP decreased from a median of 0.32 μg/L with a 2 mL sample volume to 0.16 μg/L with a 5 mL volume, with similar trends for BEHP, MBzP, and MEHP. However, the best option seems to be the use of 2 mL of sample, because although when working with 5 mL the target analyte concentration increased and therefore the sensitivity, much matrix effect was observed, with values that highly increased, like the case of DEP, MBP, or 4-HB, or even higher in the case of MEP.

When looking to the comparison of the three dilution volumes used, slight differences were observed. Working with extracts of 100 μL resulted in signal modification for all the compounds between −68 and 99%. When samples were diluted to 500 μL, the matrix effect was substantially reduced to the point where it can be considered negligible (−20% < ME% > 20%) for most of the analytes, except for 3,4-DHB, which presents a signal enhancement of 23%. Finally, the dilution to 1000 μL did not result in significant improvements in matrix effects and could potentially complicate future quantification due to decreased analyte concentration. Therefore, a final extraction volume of 500 μL was selected as the optimal working volume.

### Analytical method performance

A validation study assessing the selectivity, linearity, sensitivity, reproducibility, repeatability, and matrix effect of the new analytical method has been carried out. A summary of the validation outcomes is provided in Table 1 and Table 2.

**Table 2:** Recovery rates (%) and inter- and intra-day precision (RSD%) of the extraction procedure for the analysed compounds

				Precision			
		Recovery		Inter-day		Intra-day	
		Low level	High level	Low level	High level	Low level	High level
**OPEs**	BDCIPP	66.9	73.8	5.61	4.41	4.09	4.47
	DBP	46.0	48.6	4.14	7.00	5.61	15.7
	BBOEP	73.3	86.8	5.21	9.12	19.7	7.56
	DEP	90.3	89.4	9.32	5.49	11.7	17.7
	BEHP	88.4	93.4	3.57	4.27	15.0	9.48
**Phthalates**	MMP	93.9	86.3	2.40	4.06	13.2	4.97
	MEP	94.5	81.9	11.6	10.7	10.8	13.0
	MBzP	46.1	87.6	25.5	4.38	16.4	6.43
	MBP	80.0	87.4	14.4	9.53	19.2	25.6
	MEHP	99.4	88.7	6.51	13.3	13.6	10.9
**Parabens**	4_HB	79.1	94.8	14.4	2.97	3.39	11.52
	3,4-DHB	86.1	91.3	0.57	5.09	3.00	5.42
	MetP	91.4	96.3	4.43	0.77	1.44	17.6

Selectivity was evaluated by examining the mass accuracy, calculated as the difference between the theoretical mass and the measured *m/z* values obtained from the QTOF mass spectrometer. All target analytes exhibited mass errors below 4 ppm (Table 1), consistent with the high-resolution performance expected from the QTOF instrument. This high mass accuracy, together with the different retention times, enables reliable discrimination between the analytes and closely related endogenous compounds, thereby minimizing the risk of false positives; a common concern when analyzing complex biological matrices such as urine.

Linearity was confirmed by constructing calibration curves with eight concentration points. The method shows excellent linearity with correlation coefficients (r^2^) exceeding 0.99, meeting the accepted threshold for linearity. Calibration ranges go from 0.1 to 500 ng/mL for most analytes, except for BBOEP, MBzP, and 3.4-DHB, which had linear ranges starting at 1, 50, and 1 ng/mL, respectively. Detailed r^2^ values for all analytes can be found in Table 1.

The method also exhibited high sensitivity across all target compounds. LODs were generally between 0.01 and 0.07 ng/mL, while LOQs ranged between 0.02 and 0.23 ng/mL (Table 1). MEP, MBzP, 4-HB, and MetP had slightly higher LOD values of 0.12, 0.22, 0.34, and 0.74 ng/mL, with corresponding high LOQs of 0.41, 0.72, 1.15, and 2.45 ng/mL, respectively. Despite these elevated LODs, the method remains well-suited for detecting the typical urinary concentrations of these EDCs.

**Fig. 1:**
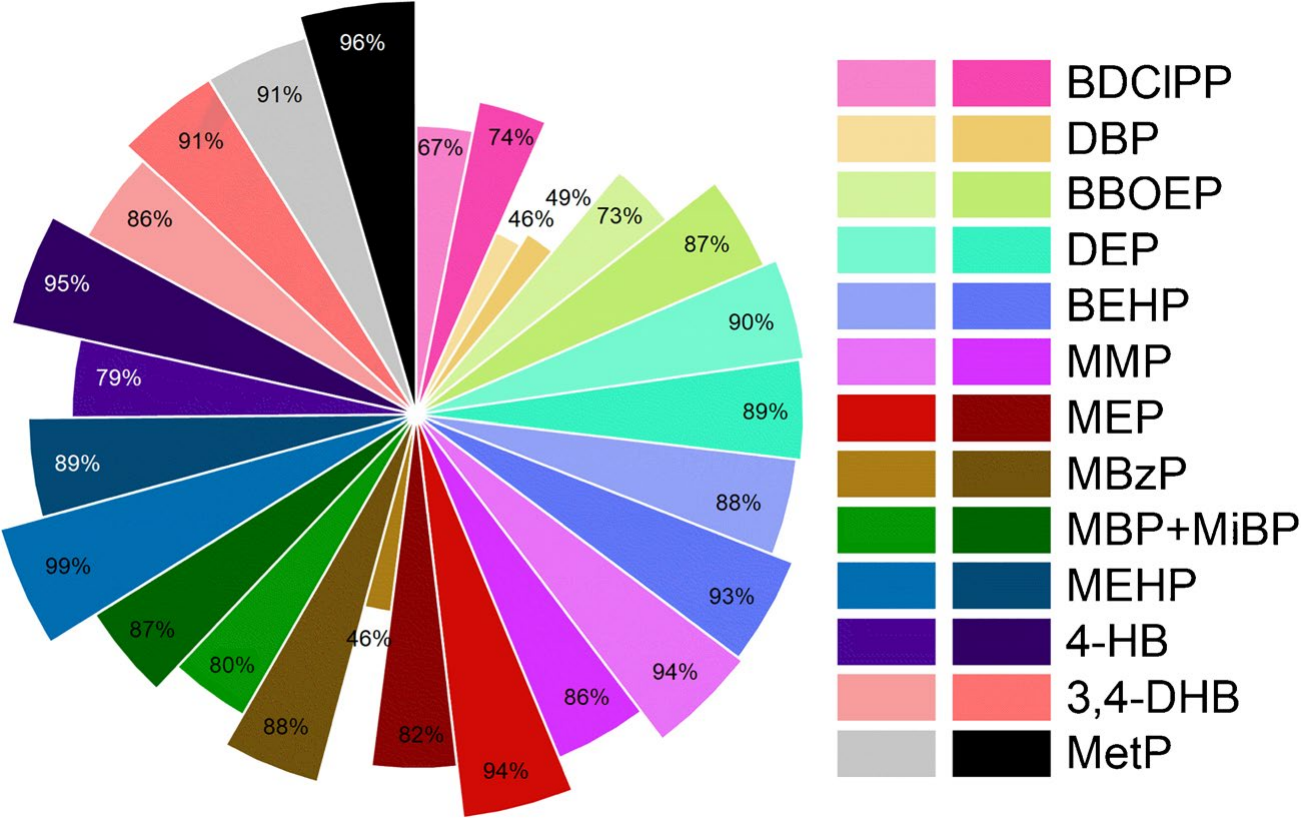
Recovery percentages at low (30 and 50 ng) and high (300 and 500 ng) concentration levels across all the metabolites analyzed

The method’s reproducibility and repeatability were assessed through recovery rates and relative standard deviation (RSD%), measured at two spiking concentrations (see the “HPLC–MS/MS analysis” section). At low spike levels, recoveries ranged from 67 to 99%, while at higher spike levels, recoveries were between 74 and 96% (Fig. 1). Two analytes, DBP and MBzP, fell just outside the ideal recovery range of 70%–120%, with DBP showing recoveries of 46% and 49%, and MBzP displaying 46% and 88% for low and high-level recoveries, respectively. However, since DBP is analyzed using its own IS, recoveries above 40% can be considered acceptable. Only MBzP at low concentrations remains below the recommended range. On the other hand, the intra-day precision presents values between 0.6% and 25%, while the inter-day precision ranges from 1.5% to 25%, indicating a strong precision of the method.

The influence of the matrix components on method performance was carefully evaluated. Significant matrix effects were observed; however, these were corrected using co-eluting IS with comparable signal modification. For analytes without an available labeled analogue, IS exhibiting similar matrix effects were selected. Based on Eq. 1, negative matrix effects were found in five compounds. Suppression levels ranged from −11% to −0.4% for BBOEP, BDCIPP, MEP, MBzP, and MBP. In contrast, the remaining eight compounds exhibited positive matrix effects, from 2 to 23%, which were generally more intense than the negative effects. The matrix effects observed were within a manageable range (−20% to + 20%), indicating minimal influence on ionization efficiency. Moreover, the signal modifications were corrected by preparing the calibration standards in the same matrix as the sample.

Finally, the null presence of the target compounds in the pure organic solvent injected after a run with the highest quality control proves that any carry-over effects observed are above the LODs.

In conclusion, all validation parameters, including selectivity, sensitivity, accuracy, and reproducibility, indicate that the method is well-suited for the biomonitoring of EDCs in urine samples.

### Comparison with existing methods

Several methods have been developed to analyze multiple EDC families in urine, employing a wide range of extraction techniques; most commonly SPE [6, 27, 33, 34] and LLE [35]. Compared to these conventional approaches, the proposed QuEChERS-based method offers significant advantages in terms of speed, simplicity, and reduced labor, making it a practical alternative for biomonitoring.

Despite its simplicity, the method showed comparable analytical performance to those reported in literature [27, 35], particularly in terms of accuracy and precision. Nevertheless, the LOD and LOQ achieved were slightly higher than those obtained in some HPLC–MS/MS-based studies [6, 23]. However, this drawback is compensated by the excellent selectivity provided by the QTOF mass spectrometer. This high selectivity is particularly important in urine analysis, where the presence of endogenous compounds can lead to mass interferences and false positives. The accurate mass measurements provided by QTOF reduce this risk significantly. Moreover, although the evaluation of matrix effects is not consistently addressed in multi-class EDC studies, some authors [6, 33, 36] reported similar or higher matrix effect values than those observed in this work. As in previous studies, both ion suppression and ion enhancement have been reported for these compounds; however, the effects remained within an acceptable range and were effectively corrected using IS.

In conclusion, while the method may sacrifice some sensitivity compared to more traditional approaches, its simplicity, speed, reduced cost, and excellent selectivity make it a more efficient and practical alternative for the simultaneous analysis of EDCs in urine analysis.

### Application of the developed method to real samples

**Fig. 2:**
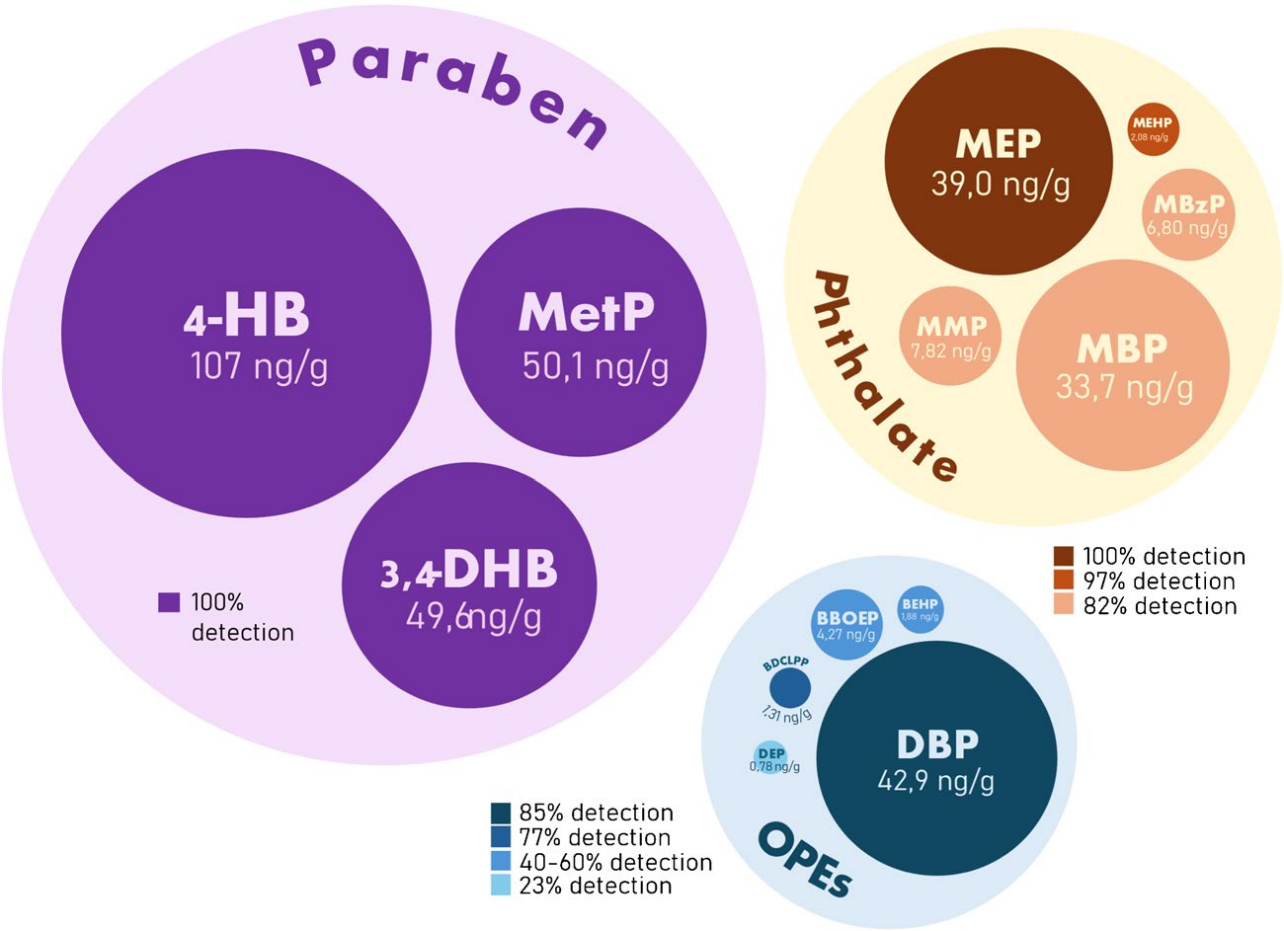
EDC metabolite concentrations (expressed in ng/g of creatinine) and detection frequencies across urine samples. (The circle’s size represents concentration levels and color intensity indicates detection frequency)

The optimized QuEChERS-LC–MS/MS methodology was successfully applied to quantify 13 EDC metabolites in 39 urine samples from the Catalan population. All targeted metabolites were detected, although their concentrations varied considerably among samples, with levels ranging from 2.10 to 183, 11.7 to 170, and 109 to 903 ng/mL of urine for OPEs, phthalates, and parabens, respectively (Table 3, and Table S3). The distribution of these metabolites across the samples is visually represented in Fig. 2.

**Table 3:** Detection frequency and concentration levels of the 13 EDCs metabolites studied in urine, expressed in ng/mL of urine (*n* = 39)

		Det. frequency	Minimum	25th percentile	Median (50th)	75th percentile	Maximum
**OPEs**	BDCIPP	77	0.73	1.01	1.31	1.83	4.67
	DBP	85	5.16	26.2	42.9	69.7	183
	BBOEP	54	0.32	1.35	4.27	11.1	19.8
	DEP	23	0.20	0.43	0.78	3.88	16.0
	BEHP	46	0.02	1.11	1.88	2.83	8.20
**Phthalates**	MMP	82	0.35	2.30	7.82	10.4	21.2
	MEP	100	5.02	23.1	39.0	52.1	93.8
	MBzP	82	1.19	4.60	6.80	9.02	13.7
	MBP	82	6.71	22.7	33.7	42.2	86.5
	MEHP	97	0.35	0.60	2.08	9.22	25.3
**Parabens**	4_HB	100	65.2	79.7	108	175	507
	3.4-DHB	100	13.8	38.5	49.6	111	308
	MetP	100	6.01	33.9	50.1	66.1	105

Among the three groups studied, parabens were the most consistently detected. All three paraben compounds were found in 100% of the samples, with total concentrations ranging from 109 to 903 ng/mL. In particular, 4-HB and 3,4-DHB—two of the main metabolites from multiple parabens—exhibited median concentrations of 108 and 49.6 ng/mL, respectively. Furthermore, the median concentration of MetP was 50.1 ng/mL, a value that aligns with those reported in investigations conducted in the USA [37], Sweden [38], and Japan [39], though higher values have been noted in studies from Spain [40] and France [41].

Phthalate metabolites were the next most abundant group, with concentrations ranging from 11.7 to 170 ng/mL. MEP was detected in all samples, Making it the most prevalent and abundant phthalate metabolite, with a median concentration of 39.0 ng/mL. Notably, the MEP levels observed here were approximately two orders of magnitude lower than those reported in a study of pregnant women in Catalonia [42] (248 ng/mL), yet they are comparable to findings from studies in China [43], Japan [44], and Sweden [45] (21.5, 21.4, and 35 ng/mL, respectively). Other phthalate metabolites—MBP, MMP, MBzP, and MEHP—displayed median values of 33.6, 7.82, 6.80, and 2.08 ng/mL, respectively, which were found to be comparable to those reported in previous literature [46, 47].

In contrast, OPE metabolites were the least frequently detected group, with concentration ranges between 2.10 and 182 ng/mL. Within this group, DBP was the most prevalent, detected in 85% of the samples and exhibiting a median concentration of 42.9 ng/mL, followed by BBOEP, which was detected in 54% of samples with a median of 4.27 ng/mL. On the other hand, DEP was the least frequently detected compound among all the EDCs studied, found in only nine samples. It is noteworthy that, aside from BDCIPP, urinary concentrations of the OPE metabolites studied have rarely been reported in the literature; only a few studies have documented the presence of DEP, DBP, and BBOEP in urine samples, although our results are largely in agreement with these limited reports [48, 49].

#### Creatinine analysis and results normalisation

Due to potential variability in individual urine dilution affecting metabolite measurements, creatinine analysis was performed on all samples following the protocol described the “Method validation” section. Subsequently, target metabolite concentrations were then normalized to nanograms per gram of creatinine to enable reliable comparisons across samples (Table S4). Creatinine levels ranged from 0.19 to 1.78 mg/mL, highlighting the substantial variation in urine dilution within the study population. Statistical analysis using the Mann–Whitney test revealed no significant differences were observed between men and women (*p* = 0.08), although women exhibited a higher median and greater variability. In contrast, significant differences in creatinine concentrations occurred between adults and teenagers (*p* = 0.0002), with teenagers exhibiting higher levels. This observation aligns with previous research demonstrating that factors such as age influence creatinine excretion.

Despite this potential limitation, creatinine normalization remains a widely used approach in biomonitoring studies, and in this work, all subsequent statistical analyses were conducted using data normalized by gram of creatinine. No significant gender differences were found for any of the three EDC groups (*p* = 0.372 for OPEs, *p* = 0.826 for phthalates, and *p* = 0.420 for parabens). However, significant differences were observed between teenagers and adults for OPEs (*p* = 0.02) and parabens (*p* = 0.00003), with the most pronounced differences noted in the paraben group.

## Conclusions

A rapid analytical methodology based on QuEChERS extraction followed by an LC–MS/MS analysis has been developed for the simultaneous determination of five OPEs, five phthalates, and three paraben metabolites in urine samples. This work represents the first application of QuEChERS sample preparation to this matrix, offering a cost-effective and time-efficient alternative to traditional SPE techniques. The extraction methodology was optimized to balance high sensitivity with minimal matrix interference. The subsequent validation demonstrated a high reproducibility, accuracy, and sensitivity of the present method, with low limits of detection and quantification that enable ultra-trace analysis of the target EDCs. Finally, the developed method was successfully applied to 39 urine samples from the Catalan population, detecting all three families of EDCs in 100% of the samples. These findings reveal significant contamination and emphasize the necessity of urine biomonitoring for these compounds. In this context, this innovative methodology is proposed as a valuable tool for comprehensive exposure assessment, as it allows the simultaneous analysis of multiple EDC families (OPEs, phthalates, and parabens) in a single procedure, optimizing sample collection, processing time, and analytical costs.

## Supplementary Information

Below is the link to the electronic supplementary material.

Supplementary Material 1 (DOCX 477 KB)

## Data Availability

Data are available on reasonable request.
