# Supplementary material for: QuEChERS analytical approach for monitoring endocrine-disrupting chemicals in human urine
Source: Anal Bioanal Chem. 2025 Sep 24;417(26):6017–27. doi: 10.1007/s00216-025-06099-1 (PMC12532665; doi:10.1007/s00216-025-06099-1)
Supplement: Supplementary file 1 — Supplementary Material 1 (DOCX 477 KB) [file 216_2025_6099_MOESM1_ESM.docx]

***Supplementary information***

**QuEChERS Analytical Approach for Monitoring Endocrine-Disrupting Chemicals in Human Urine**

S. Callejas-Martos*, E. Eljarrat*

Environmental and Water Chemistry for Human Health (ONHEALTH), Institute of Environmental Assessment and Water Research (IDAEA)-CSIC, Jordi Girona 18-26, 08034 Barcelona (Spain)

*Corresponding authors. E-mail address: scmqam@cid.csic.es, ethel.eljarrat@idaea.csic.es

**Table S1.** Parent compounds and their associated target metabolites of the analysed EDCs in this study.

| **Parent Acronym** | **Parent compound** | **Metabolite Acronym** | **Chemical name** | **Chemical Structure** | **Molecular weight [g/mol]** |
| --- | --- | --- | --- | --- | --- |
| Phthalate | | | | | |
| DMP | Dimethyl phthalate | MMP | Monomethyl phthalate | 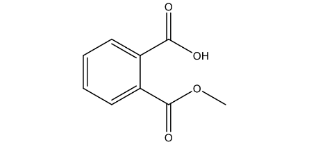 | 180,16 |
| DEP | Diethyl phthalate | MEP | Monoethyl phthalate | 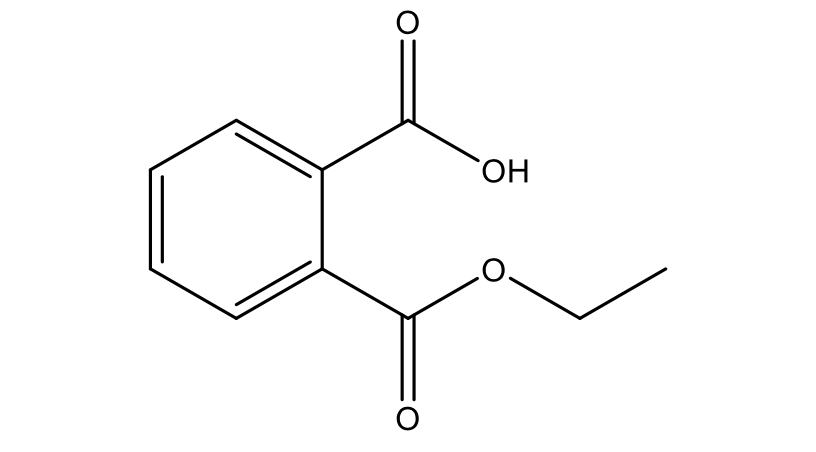 | 194,18 |
| DBP | Dibutyl phthalate | MBP | Mono-n-butyl phthalate | 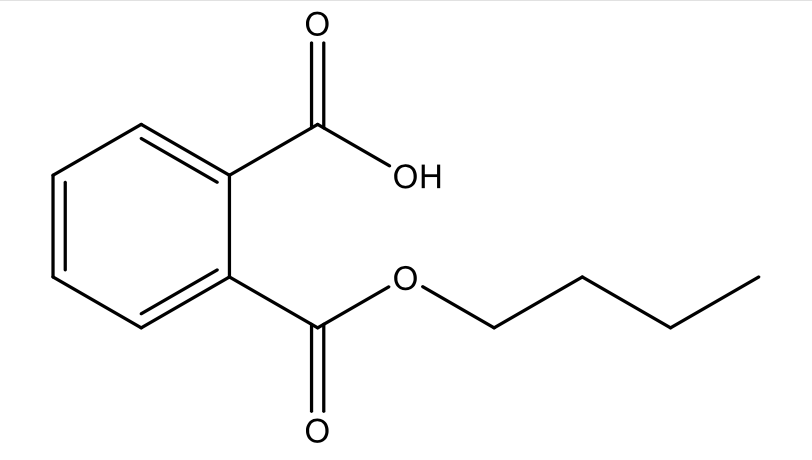 | 222,24 |
| BBzP | Benzyl butyl phthalate | MBzP | Monobenzyl phthalate | 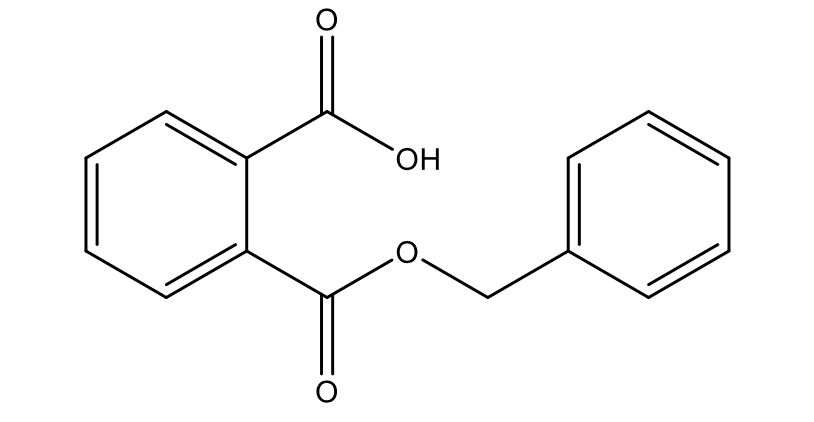 | 256,25 |
| DEHP | Bis(2-ethylhexyl) phthalate | MEHP | Mono (2-ethylhexyl) phthalate | 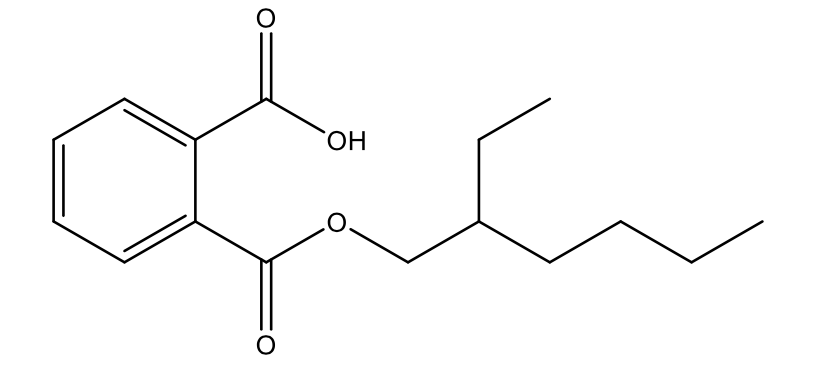 | 278,34 |
| Organophosphate ester (OPE) | | | | | |
| TDCIPP | Tris(1,3-dichloro-2-propyl) phosphate | BDCLPP | Bis(1,3-dichloro-2-propyl) phosphate | 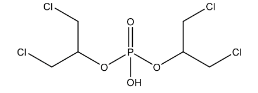 | 319,93 |
| TBP | Tributyl phosphate | DBP | Dibutyl phosphate | 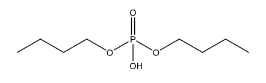 | 210,21 |
| TEP | Triethyl phosphate | DEP | Diethyl phosphate | 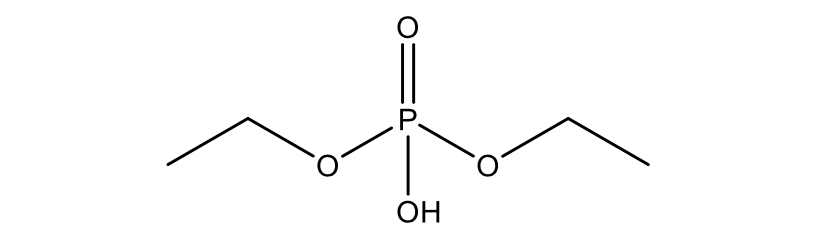 | 154,1 |
| TBOEP | Tris(2-butoxyethyl) phosphate | BBOEP | Bis(2-butoxyethyl) phosphate | 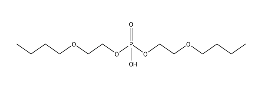 | 293,31 |
| TEHP | Tris(2-ethylhexyl) phosphate | BEHP | Bis(2-ethylhexyl) phosphate | 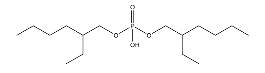 | 322,42 |
| 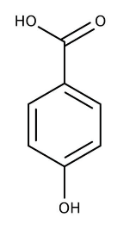Parabens | | | | | |
|  |  | 4_HB | Para-hydroxybenzoic acid |  | 138,12 |
|  |  | 3,4-DHB | 3,4-dihydroxy benzoic acid | 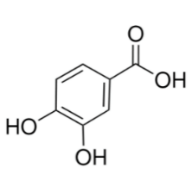 | 154,12 |
| MetP | Methyl paraben |  |  | 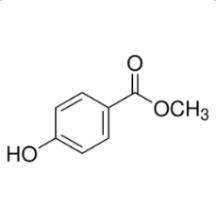 | 152,15 |

**Table S2.** Matric effect (%) of each target analyte across the different sample and final dilution volume combinations tested during the extraction method optimization. (sample volume - final dilution volume)

|  | OPEs | | | | | Phthalates | | | | | Parabens | | | |
| --- | --- | --- | --- | --- | --- | --- | --- | --- | --- | --- | --- | --- | --- | --- |
|  | BDCLPP | DBP | BBOEP | DEP | BEHP | MMP | MEP | MBzP | MBP | MEHP | | 4_HB | 3,4-DHB | MetP |
| 2-1000 | 4.4 | 8.2 | -9.3 | 7.1 | 55 | 10 | -0.4 | -2.9 | -19 | 20 | | 25 | 16 | 25 |
| 2-500 | -10 | 13 | -11 | 2.0 | 13 | 10 | -0.4 | -2.9 | -2.9 | 10 | | 2.5 | 23 | 12 |
| 2-100 | -7.0 | 24 | 5.1 | -17 | 85 | 20 | -13 | -19 | -2.9 | 30 | | 25 | 29 | 37 |
| 5-1000 | -36 | 64 | 25 | 47 | 44 | 70 | -50 | -68 | -68 | 40 | | 87 | 91 | 37 |
| 5-500 | -0.5 | 68 | 36 | 94 | 106 | 80 | -38 | -68 | -68 | 40 | | 74 | 97 | 87 |
| 5-100 | -2.5 | 70 | 47 | 72 | 96 | 90 | -50 | -68 | -68 | 30 | | 87 | 98 | 99 |

**Table S3.** EDC concentrations measured using the developed method in selected real samples, expressed in ng/mL of urine.

|  | BDCLPP | DBP | BBOEP | DEP | BEHP | ∑OPEs | MMP | MEP | MBzP | MBP | MEHP | ∑Phthalates | 4-HB | 3,4-DHB | MetP | ∑Parabens |
| --- | --- | --- | --- | --- | --- | --- | --- | --- | --- | --- | --- | --- | --- | --- | --- | --- |
| Teen_1 | 1,58 | 76,6 | 1,50 | nd | 2,18 | **81,9** | 1,06 | 42,5 | 5,41 | 42,4 | 8,83 | **100** | 73,8 | 28,7 | 60,9 | **163** |
| Teen_2 | 1,47 | 62,8 | 13,2 | 6,17 | 7,71 | **91,4** | nq | 39,0 | 9,46 | 34,6 | 5,96 | **89,0** | 81,8 | 35,4 | 63,6 | **181** |
| Teen_3 | 1,15 | 83,3 | nq | nd | nq | **84,5** | 7,46 | 38,8 | 4,72 | 25,8 | 3,81 | **80,6** | 129 | 63,2 | 92,3 | **285** |
| Teen_4 | 1,35 | 42,6 | 7,68 | 0,21 | nq | **51,8** | 8,03 | 35,5 | 7,94 | 32,5 | 0,51 | **84,5** | 70,7 | 39,9 | 92,7 | **203** |
| Teen_5 | 1,25 | 51,8 | nq | nd | nq | **53,0** | 8,44 | 71,2 | 10,6 | 40,0 | 0,35 | **131** | 79,3 | 38,5 | 48,6 | **166** |
| Teen_6 | 0,99 | 52,8 | nq | 0,65 | nq | **54,4** | nq | 35,7 | 6,13 | 40,0 | 4,17 | **86,0** | 70,7 | 42,2 | 66,1 | **179** |
| Teen_7 | 3,09 | 56,8 | 1,37 | 0,70 | 8,20 | **70,2** | nq | 45,0 | 6,51 | 72,4 | 7,94 | **132** | 110 | 33,9 | 80,3 | **225** |
| Teen_8 | 1,01 | 19,5 | 0,32 | 1,59 | nq | **22,5** | 6,20 | 93,8 | 8,34 | 51,5 | 10,4 | **170** | 108 | 44,3 | 76,8 | **229** |
| Teen_9 | 0,94 | 42,9 | 1,61 | nd | nq | **45,5** | 8,59 | 22,7 | 4,14 | 25,7 | 2,44 | **63,5** | 104 | 31,3 | 54,7 | **190** |
| Teen_10 | 1,62 | 49,5 | nq | nd | nq | **51,1** | 3,73 | 23,1 | 8,21 | 20,9 | 0,96 | **57,0** | 74,1 | 28,4 | 39,0 | **141** |
| Teen_11 | nq | 40,7 | nq | 0,85 | nq | **41,6** | 5,81 | 42,3 | 4,56 | 55,0 | nd | **108** | 90,9 | 46,8 | 69,4 | **207** |
| Teen_12 | nq | 25,3 | nq | 0,20 | 1,23 | **26,7** | 1,06 | 53,7 | 6,71 | 56,6 | 1,72 | **120** | 92,7 | 42,4 | 64,2 | **199** |
| Teen_13 | 0,73 | 31,7 | 0,72 | 0,78 | nq | **33,9** | 5,44 | 33,4 | 11,1 | 86,5 | 6,51 | **143** | 273 | 49,9 | 52,7 | **376** |
| Teen_14 | 1,15 | 106 | nd | nd | nq | **107** | 13,6 | 27,6 | 2,97 | 14,1 | 1,00 | **59,4** | 67,0 | 13,8 | 41,4 | **122** |
| Teen_15 | 2,44 | 119 | 15,9 | nd | nq | **137** | 19,8 | 62,8 | 10,2 | 23,6 | 0,61 | **117** | 83,8 | 44,0 | 28,7 | **156** |
| Teen_16 | 0,81 | 31,0 | 15,2 | nd | nq | **47,0** | 12,4 | 67,9 | 9,25 | 32,7 | 0,56 | **123** | 154 | 126 | 95,3 | **375** |
| Teen_17 | 1,95 | 48,0 | nd | nd | nq | **49,9** | 7,97 | 49,2 | 5,82 | 35,6 | 1,43 | **100** | 77,2 | 35,5 | 23,6 | **136** |
| Teen_18 | 1,24 | 115 | nd | nd | nq | **116** | 8,86 | 28,1 | 7,61 | 16,5 | 0,40 | **61,5** | 133 | 74,7 | 37,3 | **245** |
| Teen_19 | 0,81 | 38,9 | 7,89 | nd | nq | **47,6** | 10,6 | 22,0 | 2,36 | 29,5 | 0,46 | **64,9** | 183 | 157 | 86,2 | **425** |
| Teen_20 | 1,66 | 56,0 | nd | nd | 4,74 | **62,4** | 1,16 | 78,4 | 9,67 | 13,9 | 0,58 | **104** | 79,7 | 17,4 | 33,9 | **131** |
| Teen_21 | 1,39 | 61,6 | 11,9 | nd | 0,02 | **74,8** | 11,9 | 21,5 | 2,60 | 23,4 | 0,70 | **60,1** | 110 | 49,6 | 29,2 | **189** |
| Teen_22 | nd | 39,5 | nd | nd | 0,88 | **40,4** | 14,6 | 24,5 | 5,00 | 24,2 | 0,86 | **69,1** | 129 | 69,0 | 49,1 | **247** |
| Teen_23 | nd | nd | nd | nd | nq | **nd** | 9,80 | 34,3 | 7,54 | 40,0 | 0,44 | **92,2** | 87,8 | 51,6 | 50,1 | **189** |
| Teen_24 | 2,05 | 111 | nd | nd | nq | **113** | 21,2 | 46,0 | 7,21 | 50,6 | 0,60 | **126** | 182 | 111 | 59,0 | **351** |
| Teen_25 | nd | 29,6 | nd | nd | nq | **29,6** | 7,66 | 14,1 | 1,36 | 34,6 | 0,40 | **58,1** | 162 | 132 | 105 | **399** |
| Teen_26 | nd | 113 | 19,8 | nd | nq | **132** | 19,6 | 31,5 | 4,84 | 11,5 | 1,10 | **68,6** | 74,3 | 21,2 | 34,9 | **130** |
| Teen_27 | 1,19 | nd | 9,27 | nd | nq | **10,5** | 8,02 | 42,6 | 6,89 | 40,0 | 0,50 | **98,0** | 94,8 | 47,4 | 39,1 | **181** |
| Teen_28 | nd | 183 | nd | nd | nq | **183** | 5,60 | 15,2 | 1,19 | 22,4 | 2,57 | **47,0** | 109 | 50,6 | 41,3 | **201** |
| Teen_29 | 0,84 | nd | 10,3 | nd | 1,18 | **12,3** | 10,0 | 59,0 | 13,7 | 48,9 | 0,92 | **133** | 92,9 | 63,6 | 31,5 | **188** |
| Adult_1 | 4,67 | 6,47 | nd | nd | 2,91 | **14,1** | 3,26 | 52,1 | nd | 6,71 | 20,8 | **82,9** | 250 | 162 | 19,6 | **432** |
| Adult_2 | 0,84 | 5,16 | 1,33 | nd | 1,30 | **8,6** | 1,98 | 67,0 | 7,21 | nq | 18,0 | **94,2** | 207 | 178 | 20,9 | **406** |
| Adult_3 | 1,35 | nq | nd | nd | 2,28 | **3,6** | 1,14 | 69,0 | nd | nq | 24,8 | **95,0** | 333 | 166 | 33,9 | **533** |
| Adult_4 | 1,27 | nq | 0,95 | nd | 1,66 | **3,9** | 1,12 | 43,1 | 3,96 | nq | 8,09 | **56,2** | 175 | 82,2 | 14,8 | **272** |
| Adult_5 | 2,77 | 23,5 | nd | nd | 0,78 | **27,0** | nq | 19,5 | nd | 41,5 | 12,5 | **73,4** | 241 | 130 | 60,9 | **432** |
| Adult_6 | nd | 11,8 | 5,99 | nd | 1,79 | **19,6** | 1,01 | 41,6 | nd | nd | 25,3 | **67,9** | 88,0 | 44,2 | 13,6 | **146** |
| Adult_7 | nd | 25,1 | 4,27 | nd | 2,19 | **31,5** | 0,35 | 8,40 | nd | 14,9 | 13,6 | **37,2** | 108 | 61,2 | 63,2 | **232** |
| Adult_8 | 1,79 | 21,1 | 2,05 | 16,0 | 2,80 | **43,8** | nq | 5,02 | nq | nq | 6,67 | **11,7** | 325 | 279 | 52,3 | **656** |
| Adult_9 | 3,48 | 27,0 | 1,47 | nd | 1,97 | **34,0** | nq | 8,55 | 9,57 | nq | 17,5 | **35,6** | 507 | 308 | 88,2 | **903** |
| Adult_10 | 1,18 | nd | 0,39 | nd | 0,53 | **2,10** | nq | 45,3 | nd | nq | 20,8 | **66,0** | 65,2 | 38,5 | 6,01 | **110** |

**Table S4.** Detection frequency and concentration levels of the 13 EDCs metabolites studied in urine, expressed in ng/g of creatinine.

|  |  | **Det. Frequency** | **minimum** | **25th percentile** | **median (50th)** | **75th percentile** | **maximum** |
| --- | --- | --- | --- | --- | --- | --- | --- |
| **OPEs** | BDCLPP | 77 | 0,31 | 0,58 | 0,69 | 1,38 | 7,91 |
|  | DBP | 85 | 9,66 | 18,2 | 25,6 | 34,6 | 92,9 |
|  | BBOEP | 54 | 0,16 | 0,88 | 3,48 | 6,04 | 10,8 |
|  | DEP | 23 | 0,10 | 0,23 | 0,33 | 1,89 | 20,6 |
|  | BEHP | 46 | 0,01 | 0,81 | 1,72 | 3,10 | 4,92 |
| **Phthalates** | MMP | 82 | 0,24 | 2,31 | 4,21 | 6,12 | 9,25 |
|  | MEP | 100 | 5,82 | 14,0 | 17,4 | 30,4 | 176 |
|  | MBzP | 82 | 0,61 | 2,03 | 3,35 | 4,92 | 18,9 |
|  | MBP | 82 | 3,85 | 11,5 | 17,0 | 22,2 | 71,1 |
|  | MEHP | 97 | 0,14 | 0,37 | 1,41 | 6,51 | 47,1 |
| **Parabens** | 4_HB | 100 | 23,5 | 40,3 | 58,8 | 117 | 1005 |
|  | 3,4-DHB | 100 | 6,74 | 18,9 | 26,2 | 89,1 | 1101 |
|  | MetP | 100 | 8,05 | 20,5 | 30,9 | 59,6 | 127 |

**Figure S1:** Chromatogram obtained using the LC-MS/MS method employed in this study, segmented by analyzed compound families: (a) OPEs, (b) phthalates, and (c) parabens.

(c)

(b)

(a)

MPP

MEP

MEHP

BEHP

BDCLPP

BBOEP

MBP

MBzP

MetP

4_HB

3,4-DHB


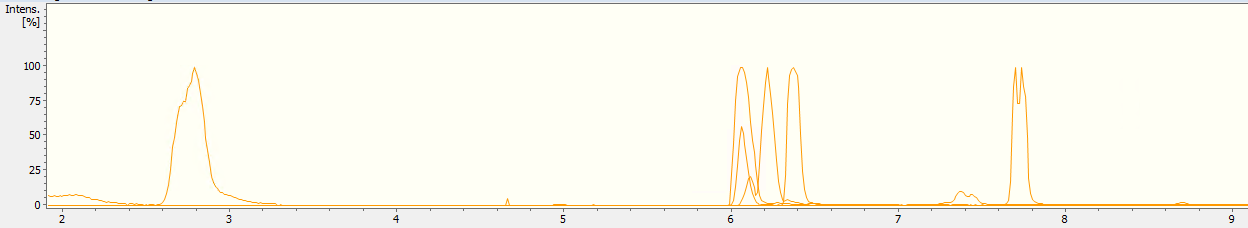

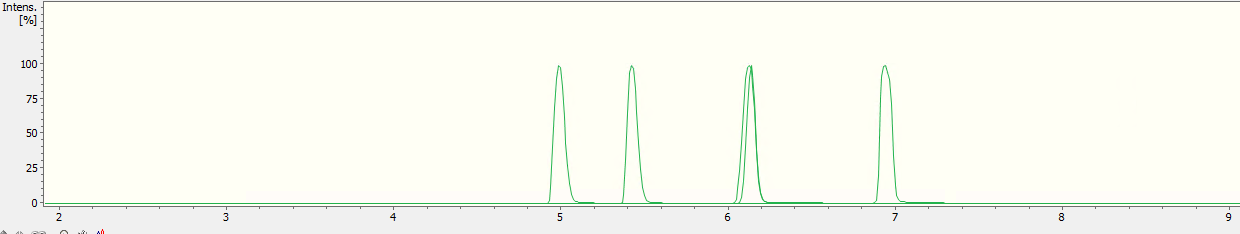

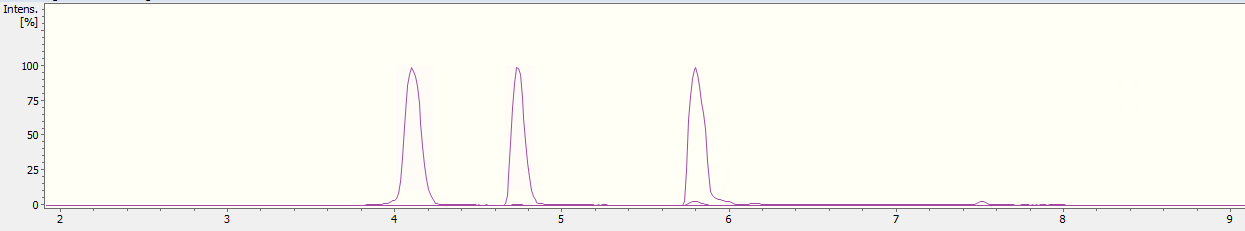


DBP

DEP
